# Antibiotics for treatment of acute exacerbation of chronic obstructive pulmonary disease: a network meta-analysis

**DOI:** 10.1186/s12890-017-0541-0

**Published:** 2017-12-12

**Authors:** Hai-Lin Zhang, Min Tan, Ai-Min Qiu, Zhang Tao, Chang-Hui Wang

**Affiliations:** 1 Department of Respiration, Shanghai Tenth People’s Hospital, Clinical Medical College, Nanjing Medical University, Extension of Middle Road 301#, Zhabei District, Shanghai 200000 Shanghai, People’s Republic of China; 2 Department of Respiration, The Affiliated Yancheng Hospital, School of Medicine, Southeast University, Yancheng, People’s Republic of China

**Keywords:** Antibiotic, Exacerbation, Chronic obstructive pulmonary disease, Meta-analysis

## Abstract

**Background:**

Acute exacerbation of chronic obstructive pulmonary disease (AECOPD) is the most common reason for the hospitalization and death of pulmonary patients. The use of antibiotics as adjuvant therapy for AECOPD, however, is still a matter of debate.

**Methods:**

In this study, we searched the PubMed, EmBase, and Cochrane databases for randomized controlled trials published until September 2016 that evaluated the use of antibiotics for AECOPD treatment. The major outcome variables were clinical cure rate and adverse effects. The microbiological response rate, relapse of exacerbation, and mortality were also analysed. A random-effect network was used to assess the effectiveness and tolerance of each antibiotic used for AECOPD treatment.

**Results:**

In this meta-analysis, we included 19 articles that assessed 17 types of antibiotics used in 5906 AECOPD patients. The cluster ranking showed that dirithromycin had a high clinical cure rate with a low rate of adverse effects. Ofloxacin, ciprofloxacin, and trimethoprim-sulfamethoxazole had high clinical cure rates with median rates of adverse effects. In terms of the microbiological response rate, only doxycycline was significantly better than placebo (odds ratio (OR), 3.84; 95% confidence interval (CI), 1.96–7.54; *p* < 0.001). There were no other significant results with respect to the frequency of recurrence or mortality.

**Conclusions:**

Our study indicated that dirithromycin is adequate for improving the clinical cure rate of patients with AECOPD with few adverse effects. Ofloxacin, ciprofloxacin, and trimethoprim-sulfamethoxazole are also recommended for disease treatment. However, caution should still be exercised when using antibiotics to treat AECOPD.

Trial Registration Not applicable.

**Electronic supplementary material:**

The online version of this article (doi: 10.1186/s12890-017-0541-0) contains supplementary material, which is available to authorized users.

## Background

Chronic obstructive pulmonary disease (COPD) is characterised by neutrophilic airway inflammation and incomplete reversible airflow restriction. Currently, approximately 5–10% adults suffer from COPD worldwide [1], especially in middle income countries. COPD is also the fourth-leading cause of death causing about 2.75 million deaths annually [2]. Acute exacerbation of COPD (AECOPD) often leads to dyspnoea, frequent cough, and a significant increase in sputum volume [3]. An exacerbation can affect the normal course of disease and severely reduce patients’ quality of life. Acute exacerbation is also the main cause of hospitalization and death of COPD patients [4], and one study showed that patients hospitalized with AECOPD who required mechanical respiratory support had a mortality rate of 40% [5].

The pathogenesis of AECOPD varies and the aetiology includes air pollution, smoking, climate change, and infection. Most AECOPD cases (80%) are caused by infection, of which 40–50% are bacterial; almost all cases involve airway inflammation. The severity of impaired lung function can vary depending on the type of pathogenic bacteria and the degree of infection [6]. Bronchodilators, mainly beta-2 receptor agonists, anti-cholinergic drugs, and theophylline, are the most commonly used agents in the treatment of AECOPD [7]. Systemic or inhaled corticosteroids and antimicrobial therapy are also important adjuvant therapies [8]. Other therapies, such as supplemental oxygen and mechanical ventilation, are also used clinically.

AECOPD quickly progresses from the onset, and the bacterial aetiology is often unclear. Generally, empirical antibiotic therapy is preferred initially when AECOPD patients are hospitalised [9]. Once the infectious bacteria are identified, specific antibiotics targeting those pathogens are used for treatment. However, at the onset of AECOPD, the physician may not be able to determine whether a patient has a bacterial infection, the type of bacteria, or the severity of infection; therefore, the type of antibiotic to be used for AECOPD treatment remains a matter of debate [10, 11].

A recent systematic review was performed to describe the therapeutic effect of moxifloxacin on AECOPD and acute exacerbation of chronic bronchitis (AECB). It was found that moxifloxacin is a safe and effective empirical agent for treatment, but this study only included a small number of AECOPD patients [12]. Another study showed that prophylactic antibiotics could effectively reduce the frequency of exacerbation; however, long-term use might increase bacterial resistance and increase the risk of adverse effects [13]. Therefore, our research systematically analysed the effect and tolerance of antibiotics for the treatment of patients with AECOPD, and a network meta-analysis was performed to directly and indirectly compare different antibiotics.

## Methods

This network meta-analysis was performed in accordance with the Preferred Reporting Items for Systematic Reviews guidelines [14].

### Data search strategy

We systematically searched the PubMed, EmBase, and the Cochrane Central Register of Controlled Trials databases using the following keywords for results published through September 2016 in the English language: chronic obstructive pulmonary disease, COPD, exacerbation, antibiotic, antimicrobial, and random*. The detail if search strategy in PubMed are presented in Additional file 1. The references of relevant reviews were also checked to ensure that no relevant studies were omitted.

### Data selection and extraction

The literature search was undertaken independently by 2 authors and any inconsistencies were settled by group discussion until a consensus was reached. A study was eligible for inclusion if it met the following criteria: 1. it was a randomised controlled trial (RCT); 2. it included patients with exacerbations of COPD but not stable COPD or prophylactic treatment for exacerbations; 3. it investigated a specific antibiotic treatment; 4. one of the outcomes included was the rate of clinical cure (success), microbiological response, relapse of exacerbations, adverse effects, or mortality. The exclusion criteria included the following: 1. it was not a RCT, such as retrospective cohort study; 2. it included another type of disease; 3. it assessed the effects of different usage strategies of only one type of antibiotic; 4. it did not include the desired results. Review articles, conference presentations, secondary research reports, letters, editorials, and basic research articles were also excluded.

We extracted the first author, publication year, country, sample size, gender ratio, experimental intervention, comparison intervention, outcome assessment, and follow-up data. We also assessed the methodological quality of the included trials using a risk of bias approach, as described by the Cochrane Collaboration [15].

In our analysis, the major efficacy outcome was clinical cure (success) rate and the major tolerance outcome was the rate of adverse effects. Secondary outcomes included microbiological response rate, relapse of exacerbations, and mortality. For the efficacy outcome analysis, we used data with the intention of treating a population comprised of randomized patients who received a study agent.

### Statistical analysis

In our analysis, we performed a pairwise meta-analysis using a random-effect model. Odds ratios (ORs) with 95% confidence intervals (CIs) were calculated to determine the size of the effect for dichotomous outcomes. We also used a random effects network meta-analysis for mixed multiple treatment comparisons [16]. To rank the treatments for each outcome, we used surface under the cumulative ranking (SUCRA) probabilities [17]. The clinical cure and adverse effect rates of each treatment are displayed as cluster-ranking plots. Comparison-adjusted funnel plots were used to determine whether small-study effects were present in our analysis [18]. All tests were two-tailed, and a *P* value of less than 0.05 was considered statistically significant. Data analyses were performed using STATA software (version 13.0; Stata Corporation, College Station, TX, USA).

## Results

### Literature search

**Fig. 1:**
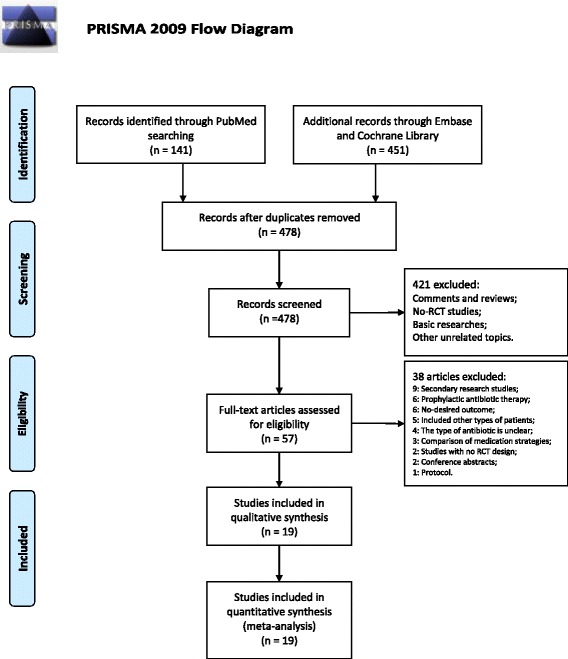
The PRISMA flowchart illustrates the selection of studies included in our analysis

In our study, 478 articles were identified after duplications were removed. After screening the titles and abstracts, 421 of these articles were excluded. The full-text of the remaining 57 articles were assessed and the following articles were excluded: secondary research studies (9), studies of prophylactic antibiotic therapy (6), studies with no desired outcome (6), studies that included other types of patients (5), studies where the type of antibiotic is unclear (4), studies that included comparison of medication strategies (3), studies without an RCT design (2), conference abstracts (2), and protocols (1). Ultimately, 19 articles, published between 1996 and 2016, that assessed 5906 patients were collected for our systematic review (Fig. 1, Table 1) [19–37]. One of the included trials contained four intervention arms [36].

**Table 1:** Characters of included studies

Author	Year	Sample size	Region	Male/female	Experimental intervention	Compare	Outcome	Follow-up#
Massimo Giusti [19]	2016	258	Italy	182/76	Levofloxacin	Prulifloxacin	Clinical cure rates; Relapse of exacerbation; Mortality	1Y
Chin Kook Rhee [20]	2015	345	Korea	312/33	Zabofloxacin	Moxifloxacin	Clinical cure rates; Microbiological response; Adverse effects	36D
Marjolein Brusse-Keizer [21]	2014	35	Netherlands	21/14	Amoxicillin-clavulanic	Placebo	Clinical cure rates; Relapse of exacerbation	4 M
Sevim Uzun [22]	2014	92	Netherlands	NA	Azithromycin	Placebo	Adverse effects	12 M
Holl Yoon [23]	2013	137	Korea	126/11	Levofloxacin	Cefuroxime	Clinical cure rates; Microbiological response; Adverse effects	14D
F.Blasi [24]	2013	357	Italy	222/135	Prulifloxacin	Levofloxacin	Clinical cure rate; Microbiological response; Adverse effects	6 M
Carl Llor [25]	2012	310	Spain	251/59	Amoxicillin-clavulanic	Placebo	Clinical cure rate; Microbiological response; Adverse effects	1Y3M
Robert Wilson [26]	2012	1492	Multicentre	833/659	Moxifloxacin	Amoxicillin-clavulanic	Clinical cure rate; Adverse effects	8 W
Semir Nouira [27]	2010	170	Tunisia	155/15	Trimethoprim-sulfamethoxazole	Ciprofloxacin	Clinical cure rate; Adverse effects; Mortality	6 M
Johannes Daniels [28]	2010	223	Netherlands	133/90	Doxycycline	Placebo	Clinical cure rate; Microbiological response	30D
Carl Llor [29]	2009	137	Spain	109/28	Amoxicillin	Amoxicillin-clavulanic	Clinical cure rate; Adverse effects	30D
Mara Rubia Andre-Alves [30]	2007	102	Brazil	59/43	Azithromycin	Amoxicillin	Clinical cure rate; Adverse effects	30D
Patrick Petitpretz [31]	2007	689	France	476/109	Levofloxacin	cefuroxime	Clinical cure rates; Adverse effects	6 M
H.Lode [32]	2004	511	Germany	277/227	Levofloxacin	Clarithromycin	Clinical cure rate; Microbiological response; Adverse effects	52 W
Ilknur Basyigit [33]	2004	30	Turkey	30/0	Clarithromycin	Placebo	Adverse effect	14D
Richard Castaldo [34]	2003	86	U.S	38/48	Dirithromycin	Azithromycin	Clinical cure rate; Adverse effects	35D
Semir Nouira [35]	2001	93	Tunisia	84/9	Ofloxacin	Placebo	Clinical cure rate; Adverse effects	26D
S.Umut [36]	1999	106	Turkey	91/15	Azithromycin	Ampicillin-sulbactam	Clinical cure rate	10D
					Ciprofloxacin	Cefaclor		
L.Allegra [37]	1996	733	France	489/244	Sparfloxacin	Amoxicillin-clavulanic	Clinical cure rate; Adverse effects	24D

#: *D* day, *W* week, *M* Month, *Y* year

### Study characteristics

**Fig. 2:**
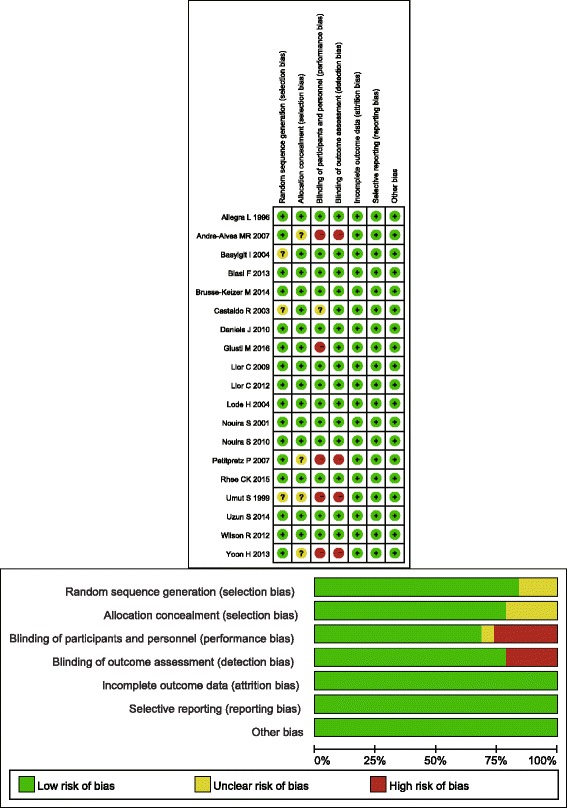
Graph of the bias risk of each study included

In the included studies, the most common research region was Europe. Studies were also conducted in Asia, North America, and Africa. There were more men than women in the study population, although one study did not mention the ratio of men to women. The following 17 antibiotics were included in our analysis: amoxicillin, amoxicillin-clavulanic acid, ampicillin-sulbactam, azithromycin, cefaclor, cefuroxime, ciprofloxacin, clarithromycin, dirithromycin, doxycycline, levofloxacin, moxifloxacin, ofloxacin, prulifloxacin, sparfloxacin, trimethoprim-sulfamethoxazole, and zabofloxacin. The shortest follow-up time was 10 days and the longest was 1 year 3 months. All the included studies were RCTs and 4 studies were open-label [23, 30, 31, 36]. Overall, the quality of the studies was ideal (Fig. 2).

### Results of network meta-analysis

**Fig. 3:**
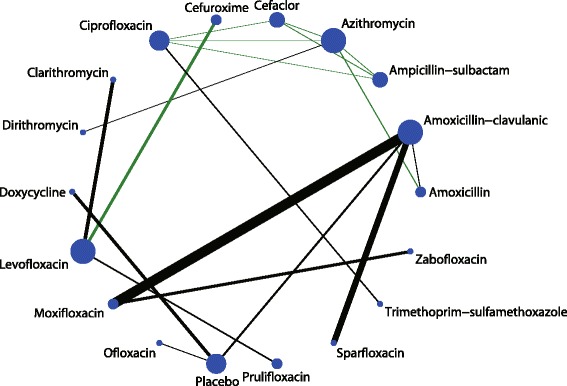
Network of comparisons for the clinical cure rate included in the analysis

**Fig. 4:**
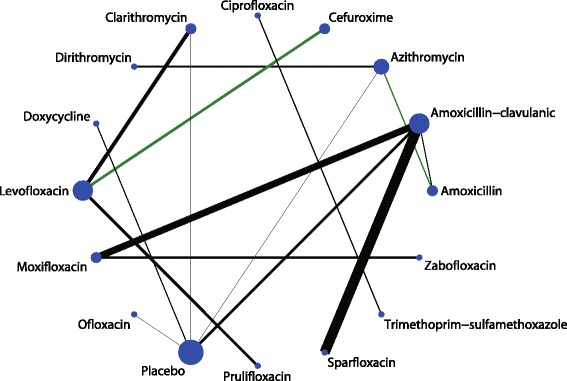
Network of comparisons for the adverse effects included in the analysis

In the network meta-analysis, the eligible comparisons of clinical cure rates are presented in Fig. 3, which are predominantly pairwise comparisons of different drug treatments for AECOPD. The figure weighs the nodes according to the number of studies that evaluated each treatment; the edges are weighed according to the precision of the direct estimate, and the edges are coloured based on the average bias level for each pairwise comparison with respect to double-blinding. Of all the comparisons, only azithromycin was directly compared with 5 other active drugs. Amoxicillin-clavulanic acid and ciprofloxacin were directly compared with 4 other drugs including a placebo. For tolerability, the eligible comparisons of adverse effects are presented in Fig. 4. Only placebo was directly compared with 5 other active drugs, and amoxicillin-clavulanic acid was directly compared with 4 other drugs including placebo.

The league table of the network for clinical cure rate assesses the treatments according to their relative effects and is shown in Additional file 2: Table S1. In terms of efficacy, ofloxacin was significantly better than both doxycycline (logOR, 2.05; 95% CI, 0.26–3.83) and placebo (logOR, 2.39; 95% CI, 0.95–3.83). Results for the adverse effects are shown in Additional file 3: Table S2. Dirithromycin was significantly better than moxifloxacin (logOR, −2.15; 95% CI, −3.71– -0.58), prulifloxacin (logOR, −2.86; 95% CI, −5.39– -0.33), sparfloxacin (logOR, −1.67; 95% CI, −3.21– -0.13), and zabofloxacin (logOR, −2.16; 95% CI, −3.88– -0.44). Placebo was significantly better than moxifloxacin (logOR, 0.99; 95% CI, 0.17–1.81).

**Fig. 5:**
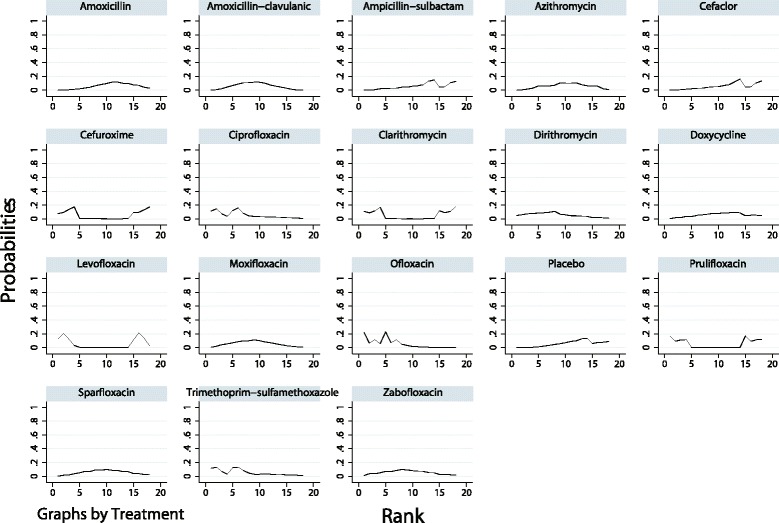
The cumulative ranking plots based on the estimation from SUCRA probabilities of the clinical cure rate

**Fig. 6:**
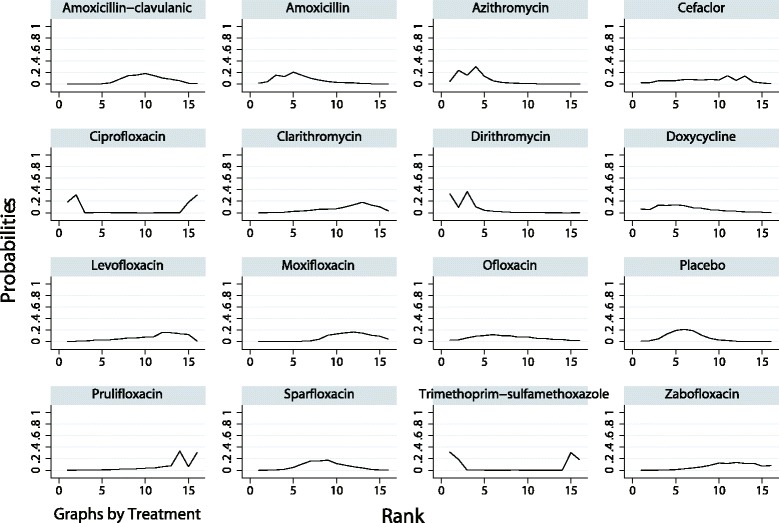
The cumulative ranking plots based on the estimation from SUCRA probabilities of adverse effects

**Fig. 7:**
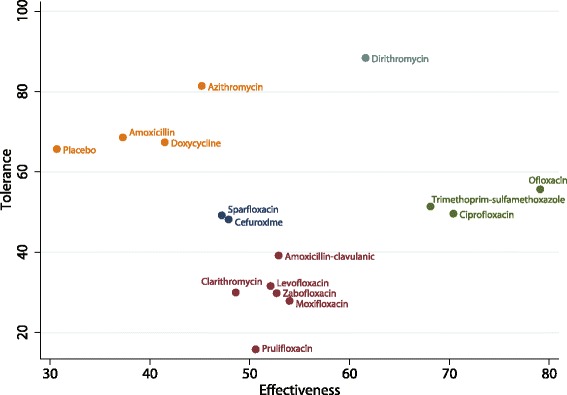
The clustered ranking plot for clinical cure rates and adverse effects

The ranking of treatments based on SUCRA probability scores is presented in Fig. 5. In terms of efficacy, ofloxacin (79.1%) was the most likely to be the best antibiotic in AECOPD treatment followed by ciprofloxacin (70.4%) and trimethoprim-sulfamethoxazole (68.1%). In terms of tolerability, dirithromycin (88.4%) was most likely to be the best drug followed by azithromycin (81.4%) and amoxicillin (68.6%; Fig. 6). After we performed a comprehensive analysis of the efficacy and tolerability, the cluster ranking showed that dirithromycin had a high clinical cure rate with a low rate of adverse effects. Ofloxacin, ciprofloxacin, and trimethoprim-sulfamethoxazole had high clinical cure rates with median rates of adverse effects (Fig. 7). There was no publication bias in the comparison-adjusted funnel plot (Additional file 4: Figure S1).

A traditional meta-analysis was also performed for the microbiological response rate, frequency of recurrence, and mortality. Only the microbiological response rate of doxycycline was significantly better than placebo (OR, 3.84; 95% CI, 1.96–7.54; *P* < 0.001) (Additional file 5: Figure S2). There were no other significant results in the frequency of recurrence (Additional file 6: Figure S3) or mortality rates (Additional file 7: Figure S4).

## Discussion

In this study, we performed a network meta-analysis to analyse antibiotic treatment in patients with AECOPD. We included several antibiotics in the analysis, including amoxicillin, amoxicillin-clavulanic acid, ampicillin-sulbactam, azithromycin, cefaclor, cefuroxime, ciprofloxacin, clarithromycin, dirithromycin, doxycycline, levofloxacin, moxifloxacin, ofloxacin, prulifloxacin, sparfloxacin, trimethoprim-sulfamethoxazole, and zabofloxacin. We found that dirithromycin had a high clinical cure rate with a low rate of adverse effects. Ofloxacin, ciprofloxacin, and trimethoprim-sulfamethoxazole had high clinical cure rates with median rates of adverse effects. Furthermore, the results of the traditional meta-analysis showed that doxycycline therapy significantly improved microbiological response rates better than placebo; however, there were no significant differences in recurrence or mortality.

The clinical diagnosis of AECOPD relies on manifestations that include a sudden change in the patients’ symptoms. Clinical and laboratory tests can rule out other diseases with the same symptoms [38], however, there is still no single biomarker for the diagnosis and assessment of AECOPD. The most common cause of AECOPD is an upper respiratory infection caused by an increase in the airway bacterial load or the emergence of a new bacterial strain [39]. In addition, viral infections, air pollution, and some unidentified pathogens can also cause AECOPD [40, 41]. The main therapeutic strategy is to reduce the severity of an exacerbation, with oxygen therapy and bronchial dilation being the initial treatments [42]. Glucocorticoids and antibiotics can shorten recovery time, improve lung function and hypoxia, and reduce early recurrence and treatment failure.

Dirithromycin belongs to the macrolide family and has a similar pharmacological activity and clinical efficacy as azithromycin, although it does not use the same metabolic pathway [43]. Dirithromycin undergoes spontaneous hydrolysis to form erythromycyclamine with the same biological activity, but it does not pass through the hepatic microsomal enzyme system. Dirithromycin is more stable than erythromycin under acidic conditions and has stronger antibacterial action against erythromycin-resistant bacteria. In drug concentration studies in AECOPD patients, dirithromycin had a higher concentration at the site of infection compared with erythromycin on standard application [44]; this suggests that dirithromycin has a greater ability to prevent bacterial superinfection with viral infection. In addition, dirithromycin did not affect the steady-state pharmacokinetics of theophylline [45]. In our results, ofloxacin, ciprofloxacin, and trimethoprim-sulfamethoxazole had high clinical cure rates with median rates of adverse effects. Ofloxacin and ciprofloxacin are third generation quinolones and synthetic antibacterial agents that primarily affect bacterial DNA, causing irreversible chromosomal damage [46]. Ofloxacin and ciprofloxacin act mainly on Gram-negative and Gram-positive bacteria, except for *Staphylococcus aureus,* and have no cross-resistance with other antibiotics; this restricts the efficacy of these drugs in bacterial superinfection after a viral infection. Moreover, a recent systematic review that analysed the effect and safety of moxifloxacin found that it may be a promising and safe alternative for the empirical treatment of AECB and AECOPD [12]. However, our results showed that moxifloxacin only had a moderate efficacy and lower tolerance compared with the efficacy and tolerance of other drugs. This may be because the comparisons of moxifloxacin versus placebo were based on indirect evidence, and the number of studies correlating the different drugs was not balanced. Trimethoprim-sulfamethoxazole is a sulphonamide and is generally a first-line antibiotic for the treatment of AECOPD; however, with its increased use, drug resistant strains and treatment failure rates have gradually increased [47]. In addition, the findings of this study suggest that several antibiotics including levofloxacin, moxifloxacin, and clarithromycin have less efficacy and lower tolerance; however, these findings are based on a small number of studies and the comparisons with other drugs were not statistically significant. These results should therefore be verified in future large-scale direct comparison trials. Finally, we noted that the tolerance of placebo was better than other drugs, which might be due to uncontrolled confounders and the nocebo effect.

A previous study suggested that COPD patients who received azithromycin for longer than 1 year had a significantly lower risk of exacerbations and a higher quality of life; however, this regimen also caused hearing loss in a small percentage of patients [48]. Furthermore, we noted that while antibiotics are effective for patients with AECOPD, they might also increase the risk of adverse effects, including hearing loss and liver, kidney, and nervous system problems. These medications may also cause allergic reactions and anaphylaxis [49]. In addition, most bacteria in the human body are beneficial, and long-term use of antibiotics will cause dysbacteriosis. The abuse of antibiotics also wastes medical resources, and it accelerates the development of super-resistant bacteria [50]. Therefore, future large-scale studies should be conducted to explore the optimal duration of antibiotic use in preventing COPD exacerbations.

Many studies have utilized procalcitonin as a biomarker of bacterial infection to guide the use of antibiotics; this is because procalcitonin is released during bacterial infections but not during viral infections or non-infectious inflammation. The result of a systematic review showed that procalcitonin-guided treatment for patients in the intensive care unit allowed for a more judicious use of antibiotics compared with standard therapy [51]. An RCT showed that there was no difference in the 6-month exacerbation and re-hospitalization rates when the treatments were compared with and without procalcitonin guidance. This study also indicated that three-day antibiotic usage had a good therapeutic effect in patients with low levels of serum procalcitonin [52]. Therefore, shortening the time of antibiotic use using procalcitonin levels could be very valuable.

Aside from antibiotic therapy, other treatments also have a crucial role in AECOPD treatment. Glucocorticoids can accelerate patient recovery, improve lung function and hypoxia, and reduce treatment failure rates; however, they might not be effective in critically ill patients [53]. Beta-receptor agonists are the first choice for bronchial dilation, and can improve clinical symptoms and lung function; long-term usage could also reduce the frequency of acute exacerbation and overall mortality. Furthermore, beta-receptor agonists are often combined with inhaled corticosteroids, which yields an even better treatment effect [54, 55]. In addition, N-acetylcysteine can be used for the treatment of COPD or chronic bronchitis to prevent exacerbations [56], and tiotropium can significantly reduce the frequency of exacerbations but not hospitalizations [57].

In the early treatment of patients with AECOPD, empirical antibiotic selection is very important for patient recovery. This network meta-analysis gives us a more comprehensive understanding of the efficacy and tolerability of these drugs; it also allow us to perform indirect comparisons between drugs. Although publication bias did not significantly impact the outcome, it should be noted that different treatment strategies, such as differences in dosages and duration of use, could impact the overall results. Bacterial antibiotic resistance also varies with time and place. Thus, the above factors need to be carefully considered when using antibiotics in the clinical setting. A comparison between traditional and network meta-analysis was not conducted, because the summary results were not consistent. Moreover, traditional meta-analyses only provide direct evidence evaluating the treatment effect of each type of antibiotic compared with placebo, whereas numerous studies provide indirect comparisons of different type of antibiotics.

There are still several limitations of this study. First, our analysis was performed on a study level, not on an individual level. Second, there were few RCTs using a single medication, which may have reduced the reliability of the meta-analysis. Third, there were other factors that could have influenced the results, such as drug dosage and the duration of use. Therefore, more RCTs of antibiotic treatment for AECOPD are needed.

## Conclusions

In conclusion, our study indicated that dirithromycin has a high clinical cure rate with a low rate of adverse effects. Ofloxacin, ciprofloxacin, and trimethoprim-sulfamethoxazole also have high clinical cure rates with median rates of adverse effects. However, caution should still be exercised when using antibiotics to treat acute exacerbations of COPD.

## Additional files

Additional file 1: Search strategy in PubMed. (DOC 18 kb)

Additional file 2: Table S1. The league table of the network for the estimation of clinical cure rates of the treatments according to their relative effects. (XLSX 12 kb)

Additional file 3: Table S2. The league table of the network for the adverse effects estimates the treatments according to their relative effects. (XLSX 12 kb)

Additional file 4: Figure S1. The comparison-adjusted funnel plot for assessing the results of clinical cure rates (A) and adverse effects (B). (EPS 872 kb)

Additional file 5: Figure S2. The forest plot shows each pairwise comparison of the microbiological response rates. (EPS 685 kb)

Additional file 6: Figure S3. The forest plot shows each pairwise comparison of the frequency of recurrence. (EPS 615 kb)

Additional file 7: Figure S4. The forest plot shows each pairwise comparison of mortality. (EPS 677 kb)

## Abbreviations

AECB: Acute exacerbation of chronic bronchitis
AECOPD: Acute exacerbation of chronic obstructive pulmonary disease
COPD: Chronic obstructive pulmonary disease
RCT: Randomised control trial
SUCRA: Surface under the cumulative ranking

## References

[1] Mannino DM, Buist AS (2007). Global burden of COPD: risk factors, prevalence, and future trends. Lancet.

[2] Steiropoulos P, Tzouvelekis A, Bouros D (2008). Formoterol in the management of chronic obstructive pulmonary disease. Int J Chron Obstruct Pulmon Dis..

[3] Spagnolo P, Fabbri LM, Bush A (2013). Long-term macrolide treatment for chronic respiratory disease. Eur Respir J.

[4] Suissa S, Dell’Aniello S, Ernst P (2012). Long-term natural history of chronic obstructive pulmonary disease: severe exacerbations and mortality. Thorax.

[5] Hurst JR (2011). Exacerbation phenotyping in chronic obstructive pulmonary disease. Am J Respir Crit Care Med.

[6] Sethi S, Murphy TF (2008). Infection in the pathogenesis and course of chronic obstructive pulmonary disease. N Engl J Med.

[7] Salpeter SR, Buckley NS, Salpeter EE (2006). Meta-analysis: anticholinergics, but not beta-agonists, reduce severe exacerbations and respiratory mortality in COPD. J Gen Intern Med.

[8] Kiser TH, Vandivier RW (2015). Severe acute exacerbations of chronic obstructive pulmonary disease: does the dosage of corticosteroids and type of antibiotic matter?. Curr Opin Pulm Med.

[9] Ouanes I, Ouanes-Besbes L, Ben Abdallah S, Dachraoui F, Abroug F (2015). Trends in use and impact on outcome of empiric antibiotic therapy and non-invasive ventilation in COPD patients with acute exacerbation. Ann Intensive Care.

[10] Vollenweider DJ, Jarrett H, Steurer-Stey CA, Garcia-Aymerich J, Puhan MA (2012). Antibiotics for exacerbations of chronic obstructive pulmonary disease. Cochrane Database Syst Rev.

[11] Soto FJ, Varkey B (2003). Evidence-based approach to acute exacerbations of COPD. Curr Opin Pulm Med.

[12] Liu KX, Xu B, Wang J, Zhang J, Ding HB, Ariani F (2014). Efficacy and safety of moxifloxacin in acute exacerbations of chronic bronchitis and COPD: a systematic review and meta-analysis. J Thorac Dis.

[13] Ni W, Shao X, Cai X, Wei C, Cui J, Wang R (2015). Prophylactic use of macrolide antibiotics for the prevention of chronic obstructive pulmonary disease exacerbation: a meta-analysis. PLoS One.

[14] Moher D, Liberati A, Tetzlaff J, Altman DG (2009). Preferred reporting items for systematic reviews and meta-analyses: the PRISMA statement. PLoS Med.

[15] Higgins JP, Altman DG, Gotzsche PC, Juni P, Moher D, Oxman AD (2011). The Cochrane Collaboration's tool for assessing risk of bias in randomised trials. BMJ.

[16] White IR, Barrett JK, Jackson D, Higgins JP (2012). Consistency and inconsistency in network meta-analysis: model estimation using multivariate meta-regression. Res Synth Methods.

[17] Li D, Wang T, Shen S, Cheng S, Yu J, Zhang Y (2015). Effects of Fluroquinolones in newly diagnosed, sputum-positive tuberculosis therapy: a systematic review and network meta-analysis. PLoS One.

[18] Trinquart L, Chatellier G, Ravaud P (2012). Adjustment for reporting bias in network meta-analysis of antidepressant trials. BMC Med Res Methodol.

[19] Giusti M, Blasi F, Iori I, Mazzone A, Sgambato F, Politi C (2016). Prulifloxacin vs Levofloxacin for exacerbation of COPD after failure of other antibiotics. COPD.

[20] Rhee CK, Chang JH, Choi EG, Kim HK, Kwon YS, Kyung SY (2015). Zabofloxacin versus moxifloxacin in patients with COPD exacerbation: a multicenter, double-blind, double-dummy, randomized, controlled, phase III, non-inferiority trial. Int J Chron Obstruct Pulmon Dis..

[21] Brusse-Keizer M, VanderValk P, Hendrix R, Kerstjens H, van der Palen J (2014). Necessity of amoxicillin clavulanic acid in addition to prednisolone in mild-to-moderate COPD exacerbations. BMJ Open Respir Res.

[22] Uzun S, Djamin RS, Kluytmans JA, Mulder PG, van't Veer NE, Ermens AA (2014). Azithromycin maintenance treatment in patients with frequent exacerbations of chronic obstructive pulmonary disease (COLUMBUS): a randomised, double-blind, placebo-controlled trial. Lancet Respir Med.

[23] Yoon HI, Lee CH, Kim DK, Park GM, Lee SM, Yim JJ (2013). Efficacy of levofloxacin versus cefuroxime in treating acute exacerbations of chronic obstructive pulmonary disease. Int J Chron Obstruct Pulmon Dis..

[24] Blasi F, Schaberg T, Centanni S, Del Vecchio A, Rosignoli MT, Dionisio P (2013). Prulifloxacin versus levofloxacin in the treatment of severe COPD patients with acute exacerbations of chronic bronchitis. Pulm Pharmacol Ther.

[25] Llor C, Moragas A, Hernandez S, Bayona C, Miravitlles M (2012). Efficacy of antibiotic therapy for acute exacerbations of mild to moderate chronic obstructive pulmonary disease. Am J Respir Crit Care Med.

[26] Wilson R, Anzueto A, Miravitlles M, Arvis P, Alder J, Haverstock D (2012). Moxifloxacin versus amoxicillin/clavulanic acid in outpatient acute exacerbations of COPD: MAESTRAL results. Eur Respir J.

[27] Nouira S, Marghli S, Besbes L, Boukef R, Daami M, Nciri N (2010). Standard versus newer antibacterial agents in the treatment of severe acute exacerbation of chronic obstructive pulmonary disease: a randomized trial of trimethoprim-sulfamethoxazole versus ciprofloxacin. Clin Infect Dis.

[28] Daniels JM, Snijders D, de Graaff CS, Vlaspolder F, Jansen HM, Boersma WG (2010). Antibiotics in addition to systemic corticosteroids for acute exacerbations of chronic obstructive pulmonary disease. Am J Respir Crit Care Med.

[29] Llor C, Hernandez S, Ribas A, Alvarez C, Cots JM, Bayona C (2009). Efficacy of amoxycillin versus amoxycillin/clavulanate in acute exacerbations of chronic pulmonary obstructive disease in primary care. Int J Chron Obstruct Pulmon Dis.

[30] Andre-Alves MR, Jardim JR, Frare E, Silva R, Fiss E, Freire DN, Teixeira PJ (2007). Comparison between azithromycin and amoxicillin in the treatment of infectious exacerbation of chronic obstructive pulmonary disease. J Bras Pneumol.

[31] Petitpretz P, Chone C, Tremolieres F (2007). Levofloxacin 500 mg once daily versus cefuroxime 250 mg twice daily in patients with acute exacerbations of chronic obstructive bronchitis: clinical efficacy and exacerbation-free interval. Int J Antimicrob Agents.

[32] Lode H, Eller J, Linnhoff A, Ioanas M (2004). Levofloxacin versus clarithromycin in COPD exacerbation: focus on exacerbation-free interval. Eur Respir J.

[33] Basyigit I, Yildiz F, Ozkara SK, Yildirim E, Boyaci H, Ilgazli A (2004). The effect of clarithromycin on inflammatory markers in chronic obstructive pulmonary disease: preliminary data. Ann Pharmacother.

[34] Castaldo RS, Celli BR, Gomez F, LaVallee N, Souhrada J, Hanrahan JP (2003). A comparison of 5-day courses of dirithromycin and azithromycin in the treatment of acute exacerbations of chronic obstructive pulmonary disease. Clin Ther.

[35] Nouira S, Marghli S, Belghith M, Besbes L, Elatrous S, Abroug F (2001). Once daily oral ofloxacin in chronic obstructive pulmonary disease exacerbation requiring mechanical ventilation: a randomised placebo-controlled trial. Lancet.

[36] Umut S, Tutluoglu B, Aydin Tosun G, Musellim B, Erk M, Yildirim N (1999). Determination of the etiological organism during acute exacerbations of COPD and efficacy of azithromycin, ampicillin-sulbactam, ciprofloxacin and cefaclor. Turkish thoracic society COPD working group. J Chemother.

[37] Allegra L, Konietzko N, Leophonte P, Hosie J, Pauwels R, Guyen JN (1996). Comparative safety and efficacy of sparfloxacin in the treatment of acute exacerbations of chronic obstructive pulmonary disease: a double-blind, randomised, parallel, multicentre study. J Antimicrob Chemother.

[38] Hurst JR, Wedzicha JA (2007). What is (and what is not) a COPD exacerbation: thoughts from the new GOLD guidelines. Thorax.

[39] Sethi S, Sethi R, Eschberger K, Lobbins P, Cai X, Grant BJ (2007). Airway bacterial concentrations and exacerbations of chronic obstructive pulmonary disease. Am J Respir Crit Care Med.

[40] MacNee W (2003). Acute exacerbations of COPD. Swiss Med Wkly.

[41] Mohan A, Chandra S, Agarwal D, Guleria R, Broor S, Gaur B (2010). Prevalence of viral infection detected by PCR and RT-PCR in patients with acute exacerbation of COPD: a systematic review. Respirology.

[42] Fromer L, Cooper CB (2008). A review of the GOLD guidelines for the diagnosis and treatment of patients with COPD. Int J Clin Pract.

[43] Sharma S, Anthonisen N (2005). Role of antimicrobial agents in the management of exacerbations of COPD. Treat Respir Med.

[44] Matera MG, Tufano MA, Polverino M, Rossi F, Cazzola M (1997). Pulmonary concentrations of dirithromycin and erythromycin during acute exacerbation of mild chronic obstructive pulmonary disease. Eur Respir J.

[45] Bachmann K, Jauregui L, Sides G, Sullivan TJ (1993). Steady-state pharmacokinetics of theophylline in COPD patients treated with dirithromycin. J Clin Pharmacol.

[46] Mensa J, Trilla A (2006). Should patients with acute exacerbation of chronic bronchitis be treated with antibiotics? Advantages of the use of fluoroquinolones. Clin Microbiol Infect.

[47] Dewan NA, Rafique S, Kanwar B, Satpathy H, Ryschon K, Tillotson GS (2000). Acute exacerbation of COPD: factors associated with poor treatment outcome. Chest.

[48] Albert RK, Connett J, Bailey WC, Casaburi R, Cooper JA, Criner GJ (2011). Azithromycin for prevention of exacerbations of COPD. N Engl J Med.

[49] Bharti VK, Srivastava RS, Kumar H, Bag S, Majumdar AC, Singh G (2014). Effects of melatonin and epiphyseal proteins on fluoride-induced adverse changes in antioxidant status of heart, liver, and kidney of rats. Adv Pharmacol Sci.

[50] Ducatelle R, Eeckhaut V, Haesebrouck F, Van Immerseel F (2015). A review on prebiotics and probiotics for the control of dysbiosis: present status and future perspectives. Animal.

[51] Tang H, Huang T, Jing J, Shen H, Cui W (2009). Effect of procalcitonin-guided treatment in patients with infections: a systematic review and meta-analysis. Infection.

[52] Verduri A, Luppi F, D'Amico R, Balduzzi S, Vicini R, Liverani A (2015). Antibiotic treatment of severe exacerbations of chronic obstructive pulmonary disease with procalcitonin: a randomized noninferiority trial. PLoS One.

[53] Cheng T, Gong Y, Guo Y, Cheng Q, Zhou M, Shi G (2013). Systemic corticosteroid for COPD exacerbations, whether the higher dose is better? A meta-analysis of randomized controlled trials. Clin Respir J.

[54] Wang J, Nie B, Xiong W, Xu Y (2012). Effect of long-acting beta-agonists on the frequency of COPD exacerbations: a meta-analysis. J Clin Pharm Ther.

[55] Du Q, Sun Y, Ding N, Lu L, Chen Y (2014). Beta-blockers reduced the risk of mortality and exacerbation in patients with COPD: a meta-analysis of observational studies. PLoS One.

[56] Cazzola M, Calzetta L, Page C, Jardim J, Chuchalin AG, Rogliani P (2015). Influence of N-acetylcysteine on chronic bronchitis or COPD exacerbations: a meta-analysis. Eur Respir Rev.

[57] Van den Bruel A, Gailly J, Neyt M (2010). Does tiotropium lower exacerbation and hospitalization frequency in COPD patients: results of a meta-analysis. BMC Pulm Med.

