# Supplementary material for: Antibiotics for treatment of acute exacerbation of chronic obstructive pulmonary disease: a network meta-analysis
Source: BMC Pulm Med. 2017 Dec 12;17:196. doi: 10.1186/s12890-017-0541-0 (PMC5727987; doi:10.1186/s12890-017-0541-0)
Supplement: Supplementary file 1 — Search strategy in PubMed. (DOC 18 kb) [file 12890_2017_541_MOESM1_ESM.doc]

**Search strategy in PubMed:**

1. Lung Diseases, Obstructive/
2. exp Pulmonary Disease, Chronic Obstructive/
3. emphysema$.mp.
4. (chronic$ adj3 bronchiti$).mp.
5. (obstruct$ adj3 (pulmonary or lung$ or airway$ or airflow$ or bronch$ or respirat$)).mp.
6. COPD.mp.
7. COAD.mp.
8. COBD.mp.
9. AECB.mp.
10. or/1-9
11. antibiotics.mp.
12. amoxicillin.mp.
13. amoxicillin-clavulanic acid.mp.
14. ampicillin-sulbactam.mp.
15. azithromycin.mp.
16. cefaclor.mp.
17. cefuroxime.mp.
18. ciprofloxacin.mp.
19. clarithromycin.mp.
20. dirithromycin.mp.
21. doxycycline.mp.
22. levofloxacin.mp.
23. moxifloxacin.mp.
24. ofloxacin.mp.
25. prulifloxacin.mp.
26. sparfloxacin.mp.
27. trimethoprim-sulfamethoxazole.mp.
28. zabofloxacin.mp.
29. or/11-28
30. 10 and 28
